# A study on the transcriptional characteristics of novel HERV gene in the blood of children with Kawasaki disease and its correlation with the disease

**DOI:** 10.1128/spectrum.00189-25

**Published:** 2025-07-28

**Authors:** Yan Lu, Minli Yuan, Juan Xu, Mengxu Yi, Xingyu Mo, Ying Zhu, Shanjie Wei, Peiting Yang, Chenglin Zhou, Wen Zhang, Hongyan Lu

**Affiliations:** 1 Department of Pediatrics, Affiliated Hospital of Jiangsu University, Jiangsu University 12676, Zhenjiang, China; 2 Clinical Laboratory Center, The Affiliated Taizhou People's Hospital of Nanjing Medical University 372209, Taizhou, China; 3 Department of Microbiology, School of Medicine, Jiangsu University 12676, Zhenjiang, China; Penn State College of Medicine, Hershey, Pennsylvania, USA

**Keywords:** endogenous retrovirus, human, kawasaki disease, phylogenetic analysis

## Abstract

Human endogenous retroviruses (HERVs), integral to the human genome, are implicated in autoimmune diseases through superantigen-mediated vascular inflammation. This study examined NCBI next-generation sequencing blood transcriptome data from 62 pairs of children with acute Kawasaki disease (KD) and healthy controls. Bioinformatics analysis identified differentially expressed HERV gene sequences (|Log_2_FC| > 0.58, FDR < 0.05), revealing 825 and 5,751 distinct HERV transcriptional variations across two KD data sets (PRJNA271099 and PRJNA739210). Novel HERVs were defined by amino acid sequence similarity of <90%. HERV-K-2177 and HERV-K-3157 demonstrated 70.27% and 56.06% protein-level similarity to known HERVs, respectively. Validation with real-time fluorescence quantitative PCR on blood samples from 18 patients with KD and 10 healthy controls showed significantly elevated expression of HERV-K-2177 and HERV-K18 mRNA in patients with KD (*P* < 0.05). Phylogenetic analysis placed HERV-K-2177 and HERV-K-3157 within the HERV-K family, though at the peripheral cluster, indicating their potential as novel HERV species. These results suggest a possible association between HERV-K-2177 transcriptional expression and KD.

**IMPORTANCE:**

Human endogenous retroviruses (HERVs) are intricate retroviruses passed down through the human genome. This study identified two novel HERVs in the blood transcriptome of children with Kawasaki disease and conducted preliminary experiments to explore the correlation between HERVs and the disease. Phylogenetic analysis indicates that these viruses may represent potential new HERV species. These findings suggest a potential association between HERV transcript expression and Kawasaki disease.

## INTRODUCTION

Kawasaki disease (KD), also known as mucocutaneous lymph node syndrome, primarily affects children under the age of 5, with a male-to-female ratio of 1.5:1. It is characterized by systemic immune-mediated vasculitis targeting small- to medium-sized vessels, often involving multiple organs, and has the highest prevalence in Asian populations. KD is the leading cause of acquired heart disease in children, with coronary artery involvement persisting in 4%–6% of cases even after standard treatment. A variety of pathogens, including bacteria, viruses, Mycoplasma pneumoniae, Chlamydia, and fungi, have been associated with KD (1). Viral infections, in particular, may play a significant role in its pathogenesis. According to current literature, at least 15 viruses have been linked to KD (2). However, no direct evidence exists to confirm their causative role. Viruses are believed to contribute to the formation of superantigens, which can hyperactivate the immune system (3–6), leading to the upregulation of matrix metalloproteinases (MMPs) and other enzymes that accelerate extracellular matrix degradation and endothelial cell destruction, resulting in coronary artery lesions (7). Human endogenous retroviruses (HERVs) may activate the host immune system (8–11). Various HERVs have been detected in patients with systemic lupus erythematosus (SLE), where proteins derived from HERV genes mimic self-antigens, triggering autoimmune responses and inflammatory reactions in SLE. HERVs, remnants of ancient retroviral infections integrated into the human genome, account for approximately 8% of the human genome. These retroviruses can be activated by genetic, environmental, and other factors, potentially contributing to the development of diseases such as SLE, Alzheimer’s disease, amyotrophic lateral sclerosis, vesicourethral epithelial carcinoma, clear-cell renal carcinoma, and breast cancer (12). Studies have shown that HERV-K mRNA expression is abnormally elevated in peripheral blood mononuclear cells (PBMCs) of elderly men and smokers, correlating with increased plasma interferon-gamma (IFN-γ) levels. This association suggests that HERVs could serve as biomarkers for diagnosing prostate cancer (13) and potentially as therapeutic targets for TP53-mutant B-cell lymphomas (14), highlighting their clinical relevance. HERV-W, a member of the ancient class I HERV family, is implicated in various diseases. For instance, HERV-W is highly expressed in the blood and cerebrospinal fluid of patients with multiple sclerosis, where its expression correlates with disease progression, clinical stage, and treatment outcomes, potentially triggered by Epstein-Barr virus (EBV) or its indirect activation (15). Direct evidence also links retroviruses to schizophrenia, with differential expression of HERV-W observed in cerebrospinal fluid and brain tissue. Its environmental genes contribute to pathogenesis through several mechanisms, such as regulating brain-derived neurotrophic factors, activating C-reactive proteins, and modulating the expression of genes like 5-hydroxytryptophan receptor 4. The associated circ_0001810 has been proposed as a potential biomarker (16–20). Furthermore, HERV-W may play a role in the pathogenesis and development of type 1 diabetes through both pro-inflammatory and cytotoxic effects (21). The envelope gene of HERV-W, syncytin-1, is abnormally overexpressed in various cancers, including endometrial cancer, leukemia, lymphoma, urothelial carcinoma (UCC), and hepatocellular carcinoma, where it is involved in tumor immune escape (22, 23). In healthy individuals, HERV transcription is generally suppressed by DNA methylation. However, factors such as viral infections, drugs, and gene mutations can modulate HERV expression. Viral infections known to regulate HERVs include cytomegalovirus, which trans-activates the HERV-K and HERV-W pol genes (24), and Dengue virus serotype 2, which upregulates numerous HERV loci (25). Human immunodeficiency virus type 1 enhances HERV-K expression (26), while EBV latent cycle genes activate the HERV-K18 superantigen (27). Additionally, the hepatitis B x protein regulates HERV-W env expression via the NF-κB signaling pathway (28). HERV-W env is also highly expressed in the blood cells of patients with COVID-19 (29). Kaposi’s sarcoma-associated herpesvirus, through primary infection or latent proteins, can activate both HERV-K and the oncogene NP9 (15). Regarding drugs, caffeine and aspirin can promote the expression of HERV-W env and gag in human SH-SY5Y neuroblastoma cells (30). Gene mutations, such as those in the 3′-LTR of HERV-W env in bladder cancer, are associated with UCC, enhancing promoter activity and contributing to tumorigenesis and progression (31). Despite the established involvement of HERVs in various diseases, their potential role in KD remains unclear. To investigate this, this study employed a bioinformatics approach to analyze public databases from NCBI, comparing the expression of known HERV genes/elements in the transcriptomes of children with KD and healthy controls. This analysis aimed to identify novel HERVs, followed by transcriptional validation of these genes in blood samples from patients with KD. Phylogenetic analysis of the novel HERVs was performed to explore the potential correlation between HERVs and KD.

## MATERIALS AND METHODS

### Retrieving and analyzing public databases for HERV transcripts in children with Kawasaki disease and controls

#### Selecting and downloading a suitable transcriptome database

The National Center for Biotechnology Information (NCBI) provides the largest and most comprehensive public data platform, encompassing various sub-databases and offering nucleic acid and protein sequence data globally. Among its many resources, the Gene Expression Omnibus (GEO) database stands out for its rigorous quality control, making it a valuable tool for researchers. The GEO database offers open access to diverse and abundant data, including high-quality data sets. In this study, the GEO database was used to search for next-generation sequencing (NGS) data related to KD using the search query “kawasaki disease AND blood AND human.” The earliest NGS data available in the GEO database dates back to December 2007. Thus, data collection period was set from December 2007 to September 2024.

Data were specifically selected from the Sequence Read Archive (SRA) in the GEO database, which contained transcriptome sequencing data relevant to KD. Selection criteria included data sets involving both KD-affected children and healthy controls, with sequencing methods encompassing second-generation sequencing of total mRNA from peripheral blood, and data available in the raw fastq.gz format.

#### Upstream analysis of HERV transcriptome data

Before analyzing the differential expression of HERVs between children with KD and healthy controls, upstream analysis of the HERV transcriptome data was conducted. On a Linux system, sequencing read quality was first evaluated using FastQC. The downloaded raw sequencing reads in FASTQ format were then quality trimmed by removing adapter sequences using Trim Galore, with the options set to "-phred 33-length 35 -stringency 3". FastQC was used again to assess the quality of the trimmed reads, after which three low-quality samples were removed. For efficient analysis, STAR software was used for one-stop processing, including comparison, conversion, and alignment. The trimmed reads were mapped to the human genome (HG38 assembly), and the mapped reads in SAM format were converted to BAM format and sorted by coordinates.

Finally, HERV quantification was performed using featureCounts, with the UCSC gene annotations source and RepeatMasker data in HISAT2. The resulting raw read count matrix was then utilized as input for all subsequent analyses in this study.

#### Differential HERV expression analysis

Differential expression analysis of the gene expression matrices was conducted using the DESeq2 package in R. HERVs were ranked by *P*-value and log_2_FoldChange, with |log_2_FoldChange| > 0.58 and Padj <0.05 considered differentially expressed. This criterion was consistently applied across both data sets, and the final upregulated and downregulated gene sets from the two databases were intersected. Differential gene expression was visualized through heatmaps, volcano plots, and Venn diagrams, created using the Pheatmap and Venn packages.

### Screening the database for novel HERVs for Kawasaki disease

To identify novel HERVs in the genomes of children with KD, the downloaded data sets were first de-joined and spliced. The resulting spliced sequences were then compared using Diamond BLASTx, annotated in Megan, and extracted from retroviral sequences. These sequences were imported into Geneious Prime to predict open reading frames (ORFs) using default parameters (minimum size: 100 bp). The predicted ORFs were subsequently compared to related coding genes using the BLASTx algorithm. HERV genes with amino acid sequence similarity of less than 90% were designated as novel HERVs. To eliminate the possibility of incorporating false sequences, these novel HERVs were re-mapped to the original transcriptome database in Geneious Prime, and coverage was calculated to confirm the true expression of these sequences.

### Validation of HERV in children with Kawasaki disease

#### Blood sample collection from children with Kawasaki disease and healthy children

Peripheral blood samples and clinical data were collected from children diagnosed with KD at the Affiliated Hospital of Jiangsu University between November 2023 and February 2024. Both complete and incomplete KD cases were included in the study. The diagnostic criteria for complete KD were as follows (32): fever lasting for 5 days or more, accompanied by at least 4 of the following 5 major clinical features: (1) non-exudative conjunctival congestion in both eyes; (2) dry, red, cracked lips, a “strawberry” tongue, and diffuse erythema of the oropharynx; (3) acute non-suppurative cervical lymphadenopathy (usually >1.5 cm in diameter); (4) polymorphous skin rash, including isolated peri-cardinal erythema; and (5) erythema of palms and soles, hard edema of hands and feet during the acute phase, and membranous desquamation around fingernails or toenails during the recovery phase, with or without coronary artery involvement. The diagnostic criteria for incomplete KD included fever lasting 5 days or more (or of variable duration), and the presence of 2 or 3 of the 5 major clinical features, along with definitive echocardiographic changes in the coronary arteries. Exclusion criteria were as follows (32): (1) presence of developmental malformations or inherited metabolic diseases; (2) family history of other malignant diseases. Healthy children of similar age and sex, who underwent physical examinations at the hospital during the same period, were used as controls. A 2 mL peripheral blood sample was collected from patients with KD in a fasting state and from healthy controls within 24 h after hospital admission. The samples were anticoagulated with ethylenediaminetetraacetic acid (EDTA), transferred to 2 mL RNase-free EP tubes and immediately stored at −80°C. The study was reviewed and approved by the Clinical Ethics Committee of the Affiliated Hospital of Jiangsu University (Ethics No. KY2023K0805), and all participants provided written informed consent.

#### Real-time quantitative PCR to verify the difference in HERV expression at nucleic acid levels between children with Kawasaki disease and healthy children

Total RNA was extracted using the Trizol method, followed by removal of gDNA with the HiScriptIII 1st Strand cDNA Synthesis Kit (+gDNA wiper) and reverse transcription into cDNA. Messenger RNA (mRNA) expression levels were analyzed using quantitative real-time PCR (qRT-PCR). Novel HERV assays were performed on an Applied Biosystems 7500 real-time PCR system with AceQ SYBR-Green qPCR Master Mix (Vazyme). Glyceraldehyde 3-phosphate dehydrogenase (GAPDH) was used as the internal reference gene to normalize expression levels. Primers were designed using Geneious Prime and Primer Premier 6, adhering to strict primer design principles. After experimental screening for single-peak melting curve primers, the sequences of primers for all targets were selected as shown in Table S1. The ΔCt method was employed for relative quantification, and data were analyzed based on comparisons of ΔCt values. After data collection, control samples were included automatically, and the average CT value for the entire run was calculated. Each sample’s CT value was then compared to the average CT value of the control to determine the relative expression level of each target gene.

#### Statistical analysis

Data were collated and analyzed using SPSS 27.0.1, with statistical analysis and plotting conducted using GraphPad Prism 10. Data that followed a normal distribution were presented as mean ± standard deviation, while categorical data were expressed as counts and percentages (%). The *F*-test assessed data normality, and unpaired *t*-tests were used to compare independent groups. Statistical significance was defined as a *P*-value < 0.05.

### Phylogenetic analysis of novel HERVs

To explore the evolutionary relationship between the novel HERVs identified in this study and known HERVs, protein sequences encoded by closely related genes were compared using BLASTp in NCBI. High-similarity reference sequences were downloaded, and low-quality comparison regions were removed using MEGA11. Novel HERVs with nucleic acid sequences shorter than 200 bp were identified using BLASTn, and their positions on the chromosome were determined. Larger ORFs were located in Geneious Prime, and ORFs of greater length were selected for protein sequence comparison. The list of all protein sequences used for phylogenetic analysis was selected as shown in Table S2, with accession numbers and source databases. Phylogenetic trees of these protein sequences were constructed by incorporating similar sequences downloaded from public databases using the neighbor-joining (NJ) method in MEGA11. The phylogenetic trees were visualized and annotated using the iTOL website.

## RESULTS

### Basic information on data

Between December 2007 and September 2024, 470 results related to human KD were identified in the GEO database, with data distributed across nine countries or regions worldwide. A total of 37 public data sets were uploaded, primarily featuring blood samples, along with some data from human set cell lymphoma (REC) and human peripheral blood set cell lymphoma cells (Mino). Earlier sequencing results were mainly derived from gene chip and microarray technologies, while high-throughput sequencing data sets predominantly emerged over the past decade. The objectives of these sequencing studies included aiding in the genetic diagnosis of KD, facilitating the differential diagnosis of bacterial, viral, and other pathogens, studying gene expression patterns in children with KD during the acute and recovery phases, and identifying differences in gene expression between responders and non-responders to IVIG treatment. Although most data sets used blood samples, there were notable variations in sample processing, including some that extracted PBMCs and others that used purified white blood cells. Sequencing methods also varied, encompassing single-cell sequencing, DNA sequencing, and RNA sequencing. For this study, 62 publicly available KD child transcriptome raw sequencing data sets were downloaded from the SRA database.

### mRNA expression of known HERVs in blood samples from children with Kawasaki disease in the SRA database

**Fig 1:**
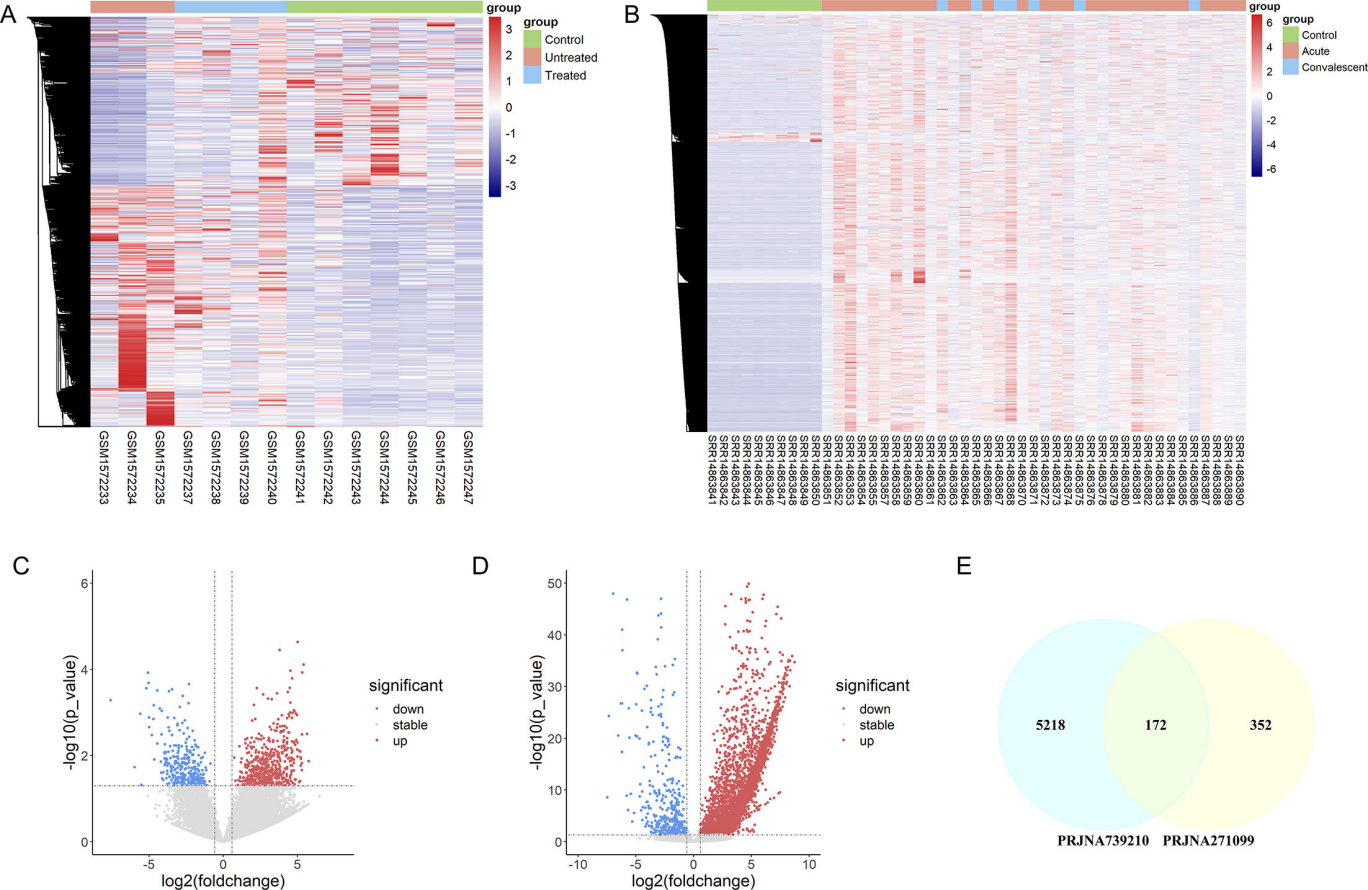
Differentially expressed HERVs in Kawasaki disease. (**A**) Heatmap of known HERV expression in the PRJNA271099 library from the blood of patients with Kawasaki disease. (**B**) Volcano plot of known HERV expression in the PRJNA271099 library. (**C**) Heatmap of known HERV expression in the PRJNA739210 library from the blood of patients with Kawasaki disease. (**D**) Volcano plot of known HERV expression in the PRJNA739210 library. (**E**) Venn diagram of HERVs co-expressed in the PRJNA271099 and PRJNA739210 libraries.

Following upstream analysis of the HERV transcriptome data, low-quality samples were excluded. Genes that showed expression across all samples (geometric mean reads per million [RPM] > 1) were retained for downstream analysis. Two KD transcriptome sequencing libraries from the SRA database were selected: one from London, UK (PRJNA271099, GSE64486) (33) and the other from La Jolla, CA, USA (PRJNA739210, GSE178491) (34). Differential expression analysis revealed a total of 825 and 5751 HERV regulatory abnormalities, comparing acute-phase whole blood RNA from patients with KD to control samples from healthy donors. The criteria for differential expression were |Log_2_FC| > 0.58 and FDR < 0.05. In the PRJNA271099 data set, 825 regulatory abnormalities were identified, including 524 upregulated HERVs and 301 downregulated HERVs. In the PRJNA739210 data set, 5751 regulatory abnormalities were detected, consisting of 5,390 upregulated HERVs and 361 downregulated HERVs. In both data sets, the number of upregulated HERVs exceeded the downregulated ones. A total of 172 HERVs were highly expressed in both data sets, identified by intersecting the upregulated HERVs from each library (Fig. 1).

### SRA database mRNA expression of novel HERV in blood samples from children with Kawasaki disease

**Fig 2:**
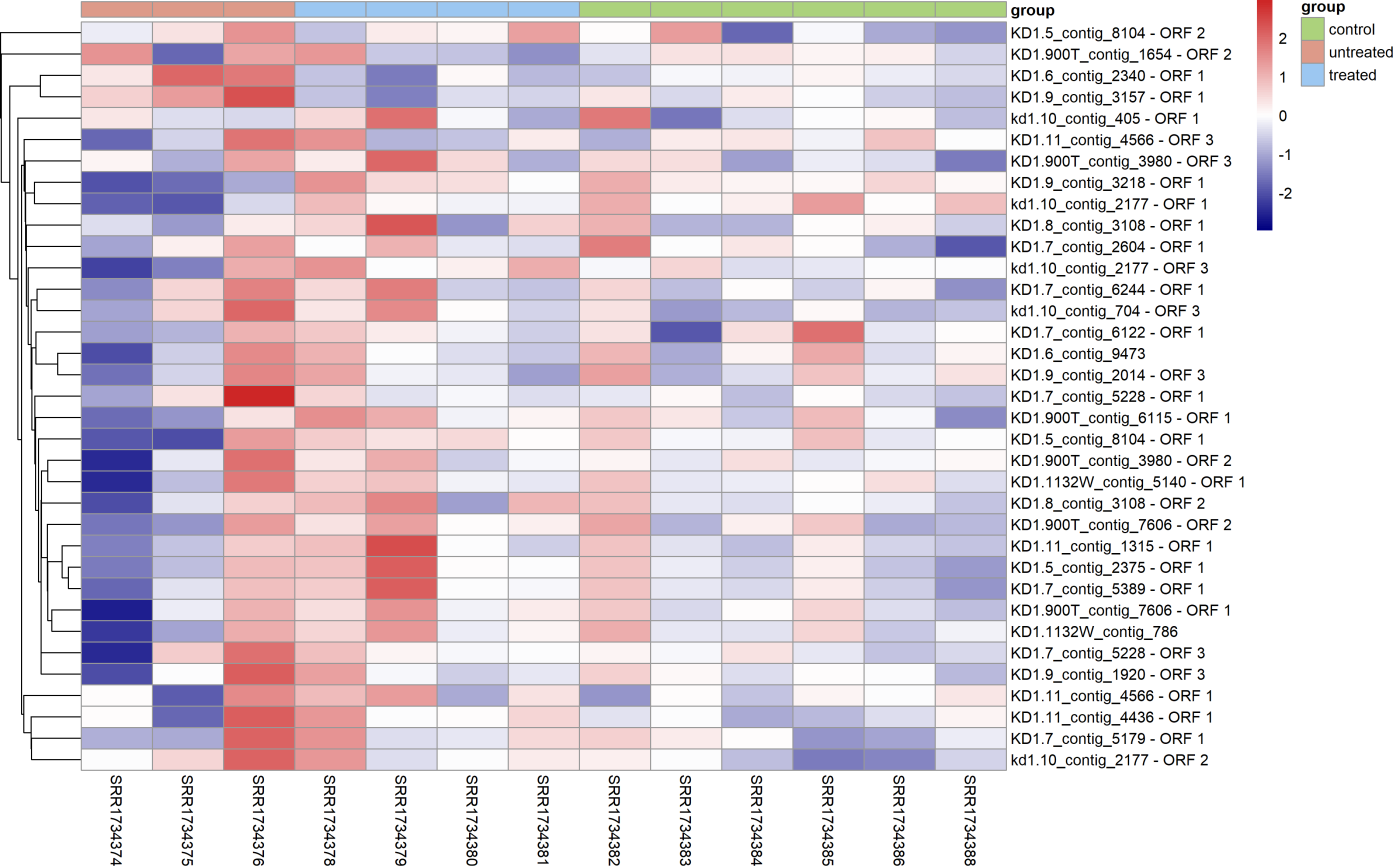
Novel HERV back-posting to the PRJNA271099 database presented as a heatmap.

Using the criterion of less than 90% amino acid sequence similarity to known viruses for determining novel HERVs, 35 novel HERVs were identified in the PRJNA271099 data set. These novel HERVs ranged in length from 100 nucleotides or more, contained complete ORFs, and exhibited sequence identity with known viral sequences ranging from 37.00% to 89.47%, suggesting the potential identification of new HERV species (Table 1). Some of these sequences were shorter than the reference genome of endogenous retroviruses (ERVs), indicating that they might represent fragments of the viral genome. Additionally, viral sequences with greater than 90% identity were predicted and compared to known HERV elements. To exclude the possibility of incorporating false sequences, the identified sequences were re-mapped to the original transcriptome database using Geneious Prime to assess the coverage. RPKM normalization was applied to calculate the read lengths, multiplying the number of original read lengths of R1 in the upper bipartite and the length of the ORF sequences to determine the true expression. The data were further scaled up and treated with log2 normalization for subsequent analysis. The expression levels of the ORFs in the original library ranged from 0.66 to 7.73, with varying degrees of read matching. However, these sequences could not be assembled into overlapping clusters of sufficient length, possibly due to differences in library construction methods or insufficient sequencing depth (Fig. 2).

**TABLE 1:** Comparison of novel HERVs in the PRJNA271099 library in NCBI^*a*^,^*b*^

No.	Name		Description	Scientific name	Max score	Total score	Query cover	E value	Per.ident	Acc. len	Chrom	Strand	Range
1	KD1.900T_contig_3980	ORF2	polyprotein	Multiple sclerosis-associated retrovirus	86.7	86.7	84%	1.00E-17	93.18%	768	Homo sapiens 12 BAC RP11-20D14 (Roswell Park Cancer Institute Human BAC Library) complete sequence	-	117,387 to 117,541
			endogenous retrovirus group K member 113 Pol protein-like	Myotis davidii	54.7	54.7	96%	2.00E-06	52.94%	1,095			
2		ORF3	polyprotein	Multiple sclerosis associated retrovirus	72	124	99%	2.00E-12	76.09%	768	Homo sapiens 12 BAC RP11-20D14 (Roswell Park Cancer Institute Human BAC Library) complete sequence	-	117,412 to 117,606
			polyprotein	Haliaeetus leucocephalus	51.2	97	99%	7.00E-11	51.06%	797			
3	KD1.900T_contig_6115		env protein	Homo sapiens	127	127	87%	5.00E-32	90.62%	584	Homo sapiens BAC clone CH17-35L3 from chromosome 16, complete sequence	+	201,107 to 201,325
			Full = HERV H_2q24.3 provirus ancestral Env polyprotein	Homo sapiens	128	128	87%	4.00E-32	90.62%	584			
4	KD1.900T_contig_7606	ORF1	protease	Homo sapiens	104	104	100%	7.00E-25	60.00%	273	Homo sapiens chromosome 5 clone CTD-2083E4, complete sequence	-	99,955 to 100,179
			endogenous retrovirus group K member 8 Gag polyprotein-like	Symphalangus syndactylus	105	105	100%	6.00E-24	60.00%	942			
5		ORF2	endogenous retrovirus group K member 8 Gag polyprotein-like	Symphalangus syndactylus	66.6	66.6	97%	8.00E-11	61.70%	942	Homo sapiens chromosome 5 clone CTD-2083E4, complete sequence	-	99,975 to 100,118
			endogenous retrovirus group K member 9 Pol protein-like	Rhinopithecus bieti	65.5	65.5	97%	2.00E-10	59.57%	323			
6	KD1.1132_contig_786		pol protein	Homo sapiens	146	146	99%	1.00E-41	80.46%	222	Homo sapiens chromosome 5 clone CTD-2083E4, complete sequence	+	100,684 to 100,945
			pol precursor	Homo sapiens	142	142	99%	4.00E-41	79.31%	135			
7	KD1.1132_contig_5140		envelope protein	Homo sapiens	97.1	97.1	97%	8.00E-22	54.00%	242	Human DNA sequence from clone RP13-179C6 on chromosome X, complete sequence	+	145,701 to 145,995
			recombinant envelope protein	Multiple sclerosis-associated retrovirus element	98.2	98.2	93%	6.00E-21	50.00%	542			
8	KD1.5_contig_2375		Gag-Pro-Pol-Env protein	Homo sapiens	82.4	82.4	96%	4.00E-16	70.37%	2,294	Human DNA sequence from clone RP11-363I22 on chromosome 1, complete sequence	+	8,966 to 9,135
			pol protein	Homo sapiens	82.4	82.4	96%	4.00E-16	70.37%	872			
9	KD1.900T_contig_1654		pol protein	Homo sapiens	46.6	46.6	97%	6.00E-04	63.89%	702	Homo sapiens chromosome 16 clone RP11-337N9, complete sequence	+	117,502 to 117,603
			Gag-Pro-Pol protein	Homo sapiens	46.6	46.6	97%	6.00E-04	63.89%	1,879			
10	KD1.6_contig_2340		putative pol protein	Porcine endogenous retrovirus	60.5	60.5	91%	3.00E-09	55.38%	125	Homo sapiens BAC clone RP11-559E14 from chromosome x, complete sequence	-	78,222 to 78,434
			Retrovirus-related Pol polyprotein from transposon 17.6	Eudyptes chrysocome	63.2	63.2	81%	4.00E-09	57.63%	1,083			
11	KD1.6_contig_9437		pro-pol-dUTPase polyprotein - murine endogenous retrovirus ERV-L	Murine endogenous retrovirus ERV-L	159	159	83%	4.00E-42	81.82%	1,182	Homo sapiens chromosome 3 clone RP11-1029M24, complete sequence	+	31,624 to 31,938
			endogenous retrovirus group K member 113 Pol protein-like	Chinchilla lanigera	154	154	83%	1.00E-40	78.41%	1,201			
12	KD1.7_contig_2604		pol protein	Mouse mammary tumor virus	56.2	56.2	97%	3.00E-08	68.42%	111	Homo sapiens chromosome 5 clone CTB-54I1, complete sequence	+	43,248 to 43,364
			endogenous retrovirus group K member 11 Pol protein-like	Pseudopodoces humilis	57.8	57.8	97%	8.00E-08	73.68%	655			
13	KD1.7_contig_5179		Endogenous retrovirus group K member 18 Pol protein	Homo sapiens	63.2	63.2	100%	1.00E-09	69.57%	812	Homo sapiens chromosome 5 clone CTD-2195H9, complete sequence	-	7,083 to 7,216
			Endogenous retrovirus group K member 11 Pol protein	Homo sapiens	62	62	95%	4.00E-09	70.45%	969			
14	KD1.7_contig_5228	ORF1	envelope glycoprotein	Plecturocebus cupreus	47.4	47.4	97%	3.00E-04	59.46%	1,691	Human DNA sequence from clone RP11-29L10 on chromosome Xq21.1-21.33, complete sequence	+	74,964 to 75,077
			endogenous retrovirus group FC1 member 1 Env polyprotein-like	Callithrix jacchus	47.4	47.4	92%	4.00E-04	62.86%	589			
15		ORF3	Gag-Pol polyprotein	Plecturocebus cupreus	56.6	56.6	97%	2.00E-07	57.78%	278	Human DNA sequence from clone RP11-29L10 on chromosome Xq21.1-21.33, complete sequence	-	74,967 to 75,102
			Pol polyprotein	Plecturocebus cupreus	53.9	53.9	97%	9.00E-07	55.56%	161			
16	KD1.7_contig_5389		pol protein	Homo sapiens	124	124	92%	3.00E-30	67.78%	702	Homo sapiens BAC clone RP11-153M7 from 4, complete sequence	+	124,279 to 124,572
			pol protein	Homo sapiens	124	124	92%	4.00E-30	67.78%	872			
17	KD1.7_contig_6122		polymerase	Homo sapiens	48.9	48.9	90%	7.00E-05	61.11%	198	Homo sapiens 12 BAC RP11-328C8 (Roswell Park Cancer Institute Human BAC Library) complete sequence	+	42,513 to 42,632
			endogenous retrovirus group K member 18 Pol protein-like	Odobenus rosmarus divergens	48.5	48.5	87%	2.00E-04	62.86%	489			
18	KD1.7_contig_6244		polyprotein	Multiple sclerosis associated retrovirus	105	105	89%	3.00E-24	86.21%	768	Homo sapiens FOSMID clone ABC11-47179300H24 from chromosome unknown, complete sequence	+	17,586 to 17,762
			endogenous retrovirus group K member 113 Pol protein-like	Haliaeetus leucocephalus	79.3	79.3	90%	7.00E-15	65.08%	797			
19	KD1.8_contig_3108	ORF1	Endogenous retrovirus group K member 25 Pol protein	Homo sapiens	74	74	52.00%	0.017509	53.57%	812	Homo sapiens BAC clone RP11-153M7 from 4, complete sequence	+	122,788 to 122,948
			Endogenous retrovirus group K member 6 Pol protein	Homo sapiens	91	91	94.00%	3.90E-05	39.22%	969			
20		ORF2	Endogenous retrovirus group K member 7 Pol protein	Homo sapiens	75	75	98.00%	0.006073	40.00%	956	Homo sapiens BAC clone RP11-153M7 from 4, complete sequence	-	122,805 to 122,926
			Endogenous retrovirus group K member 11 Pol protein	Homo sapiens	75	75	98.00%	0.005941	40.00%	954			
21	KD1.9_contig_1920		envelope glycoprotein	Plecturocebus cupreus	75.5	75.5	85%	1.00E-13	63.64%	819	Homo sapiens chromosome 19, BAC 82621 (CIT-B-139a18), complete sequence	-	19,605 to 19,797
			envelope glycoprotein	Plecturocebus cupreus	72.4	72.4	92%	1.00E-12	59.32%	850			
22	KD1.9_contig_2014		pro-pol-dUTPase polyprotein - murine endogenous retrovirus ERV-L	Murine endogenous retrovirus ERV-L	155	155	98%	5.00E-41	77.55%	1,182	Homo sapiens genomic DNA, chromosome 11q clone:CMB9-100I11, complete sequences	-	111,665 to 111,960
			endogenous retrovirus group K member 113 Pol protein-like	Chinchilla lanigera	153	153	98%	3.00E-40	75.51%	1,201			
23	KD1.9_contig_3157		Gag-Pro-Pol protein	Homo sapiens	83.2	83.2	98%	4.00E-16	56.06%	1,755	Homo sapiens PAC clone RP5-981O7 from 7q34-q36, complete sequence	-	31,075 to 31,273
			Gag-Pro-Pol protein	Homo sapiens	82.8	82.8	98%	4.00E-16	56.06%	1,879			
24	KD1.9_contig_3218		endogenous retrovirus group K member 10 Gag polyprotein-like	Aotus nancymaae	64.3	64.3	100%	7.00E-10	55.10%	531	Homo sapiens chromosome 5 clone CTD-2245E15, complete sequence	-	30,355 to 30,501
			endogenous retrovirus group K member 5 Gag polyprotein-like	Homo sapiens	56.2	56.2	100%	5.00E-07	48.98%	623			
25	KD1.10_contig_405		putative pol protein	Porcine endogenous retrovirus	82.8	82.8	94%	3.00E-18	89.58%	125	Human DNA sequence from clone RP13-147D17 on chromosome X, complete sequence	+	93,611 to 93,763
			polymerase	Melomys burtoni retrovirus	70.9	70.9	94%	3.00E-12	75.00%	939			
26	KD1.10_contig_2177	ORF1	Gag-Pro-Pol-Env protein	Homo sapiens	63.9	63.9	97%	4.00E-10	71.43%	2,294	Human DNA sequence from clone RP11-323H21 on chromosome 9q34.1-34.3, complete sequence	+	862 to 969
			Gag-Pro-Pol protein	Homo sapiens	63.9	63.9	97%	4.00E-10	71.43%	1,879			
27		ORF2	Gag-Pro-Pol-Env protein	Homo sapiens	69.7	69.7	94%	4.00E-12	70.27%	2,294	Homo sapiens chromosome 8, clone RP11-30L15, complete sequence	+	29,515 to 29,633
			Gag-Pro-Pol protein	Homo sapiens	69.7	69.7	94%	4.00E-12	70.27%	1,879			
28		ORF3	Endogenous retrovirus group K member 7 Pol protein	Homo sapiens	55.1	55.1	100%	7.00E-07	61.11%	727	Homo sapiens cDNA: FLJ23457 fis, clone HSI07266	-	797 to 904
			pol protein	Homo sapiens	55.1	55.1	100%	7.00E-07	61.11%	1,459			
29	KD1.11_contig_1315		pol protein	Homo sapiens	142	142	98%	2.00E-36	62.96%	702	Homo sapiens BAC clone RP11-434N4 from 2, complete sequence	-	100,898 to 101,224
			Endogenous retrovirus group K member 8 Pol protein	Homo sapiens	142	142	98%	3.00E-36	62.96%	956			
30	KD1.11_contig_4436		Gag-Pro-Pol-Env protein	Homo sapiens	55.5	90.5	96%	5.00E-09	95.65%	2,294	Human DNA sequence from clone RP11-404F10 on chromosome 1q23.1-24.1, complete sequence	-	84,000 to 84,120
			Gag-Pro-Pol protein	Homo sapiens	55.8	90.5	96%	6.00E-09	95.65%	1,879			
31	KD1.11_contig_4566	ORF1	pol protein	Homo sapiens	62	62	100%	2.00E-09	85.29%	702	Homo sapiens BAC clone VMRC62-286I17 from chromosome unknown, complete sequence	-	68,413 to 68,514
			pol protein	Homo sapiens	62	62	100%	2.00E-09	85.29%	702			
32		ORF3	pol protein	Homo sapiens	72.8	72.8	97%	5.00E-13	89.47%	702	Homo sapiens isolate HML-2_4 p16.3b endogenous virus HERV-K, complete sequence	-	5,203 to 5,319
			pol protein	Homo sapiens	72.4	72.4	97%	5.00E-13	89.47%	872			
33	KD1.10_contig_704		endogenous retrovirus group S71 member 1 Env polyprotein-like isoform X1	Panthera uncia	53.9	53.9	83%	4.00E-05	37.00%	602	Homo Sapiens Chromosome X clone bWXD691, complete sequence	-	46,000 to 46,358
			endogenous retrovirus group S71 member 1 Env polyprotein-like	Panthera pardus	53.9	53.9	83%	4.00E-05	37.00%	552			
34	KD1.5_contig_8104	ORF1	polyprotein	Multiple sclerosis-associated retrovirus	63.5	63.5	97%	6.00E-10	78.79%	768	Homo sapiens, clone RP11-44K6, complete sequence	+	20,804 to 20,905
			endogenous retrovirus ERV9	Homo sapiens	43.5	43.5	82%	0.003	89.29%	129			
35		ORF2	endogenous retrovirus ERV9	Homo sapiens	50.1	50.1	87%	0.00001	76.47%	129	Homo sapiens ataxin 1 (ATXN1), RefSeqGene (LRG_863) on chromosome 6	-	306079 to 306195
			polyprotein	Multiple sclerosis associated retrovirus	42.4	42.4	97%	0.022	60.53%	768			

^*a*^ The table compares the endogenous retroviruses identified and their similarity across genes, along with the chromosomal locations of these genes.

^*b*^ "+" indicates the genetic element is on the reference genome's positive strand, while "-" denotes it is on the negative strand.

### General characteristics of children with Kawasaki disease and healthy control children

In this study, 18 children with KD were included: 6 males and 12 females, with ages ranging from 1 year and 6 months to 10 years, and a median age of 4 years. The control group consisted of 10 healthy children: 3 males and 7 females, with ages ranging from 2 years to 9 years, and a median age of 6 years. There were no statistically significant differences in age and sex between the KD group and the control group (*P* > 0.05) (Table 2).

**TABLE 2:** General characteristics of children with Kawasaki disease and healthy control children

Groups	Number of examples	Sex (m/f)	Age (months)
Kawasaki disease	18	6/12	52.06 ± 31.460
control	10	3/7	73.90 ± 24.251
F		0.934	0.336
P		0.343	0.567

### Gene expression analysis of HERVs in children with Kawasaki disease and healthy children

**Fig 3:**
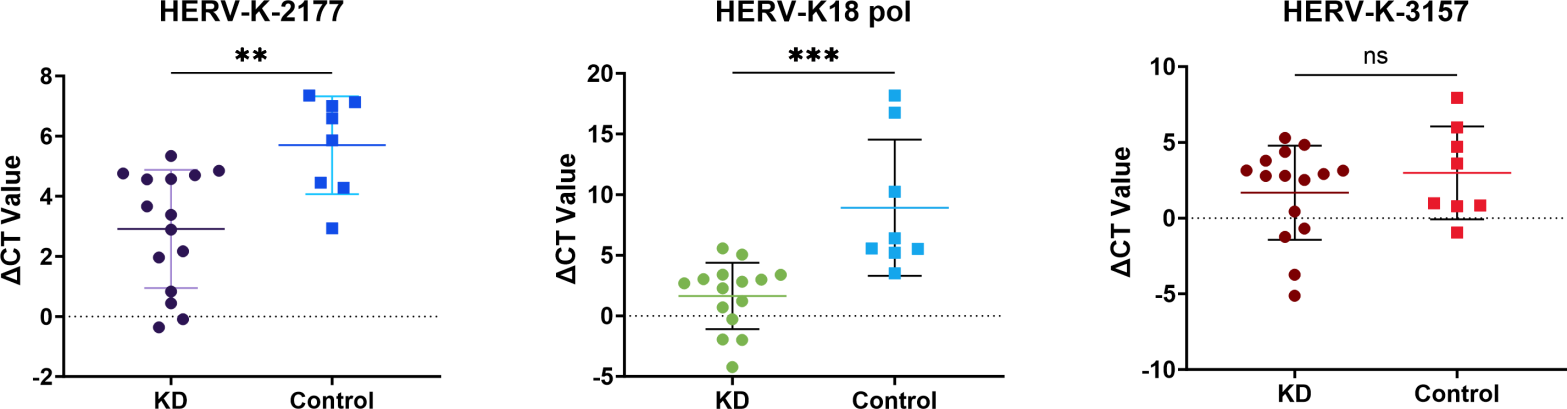
Scatter plot showing the expression of HERV-K-2177, HERV-K18 pol, and HERV-K-3157 genes in children with Kawasaki disease and controls.

In the visualization of the novel HERV expression matrix and heatmap, HERV-K-2177 (designated as *KD1.10_contig_2177 ORF2* during analysis) exhibited the highest expression of 4.99 in children with KD during both the acute and recovery phases, compared to 3.89 in the control group, indicating a significant difference. HERV-K-3157 (designated as KD1.9_contig_3157) showed a peak expression of 5.71 in children in the acute phase of KD, higher than the peak of 3.31 in controls and 2.38 in the recovery phase, suggesting an upregulation trend. Additionally, the gene later confirmed as HERV-K18 (*KD1.11_contig_4566 ORF2*) was expressed at 4.99 in the KD group, while in the control group, it was 3.89, highlighting a more pronounced difference. The highest expression in the KD group was 5.94, compared to 3.10 in the control group, reinforcing the upregulation trend. These three HERV genes were selected for primer design, with single-peak melting curves confirming specific amplification. Expression levels were validated by real-time fluorescence quantitative RT-PCR, and ΔCT values (ΔCT = CT_HERV_ - CT_GAPDH_) were compared between the two groups. The results revealed significantly higher expression of HERV-K-2177 and *HERV-K18 pol* in the peripheral blood of children with KD compared to healthy controls (*P* < 0.05). However, no significant difference in the expression levels of the novel HERV-K-3157 was observed (Table 3and Fig. 3).

**TABLE 3:** Novel HERV transcript levels in children with Kawasaki disease and healthy control children

Genetics	ΔCT values (mean ± SD)		
	KD group	Healthy control group	*P*
HERV-K-2177	2.91 ± 1.97	5.33 ± 1.89	0.0072
HERV-K-3157	1.68 ± 3.10	2.99 ± 3.07	0.3456
*HERV-K18 pol*	1.64 ± 2.74	8.92 ± 5.62	0.0004

### Phylogenetic analysis of novel HERVs

**Fig 4:**
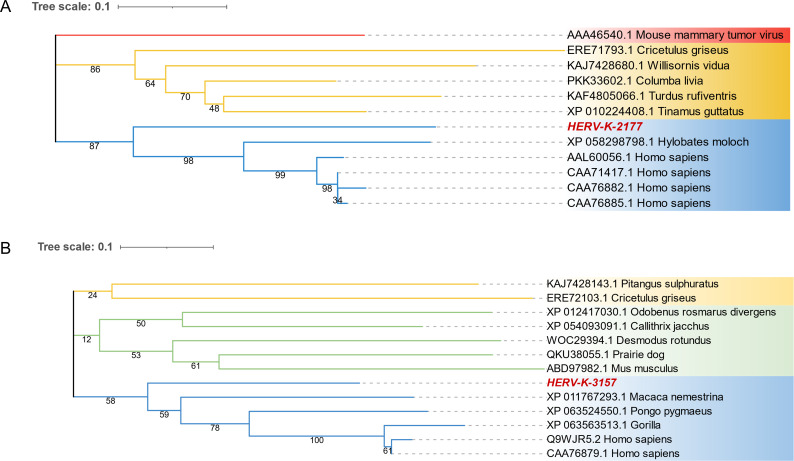
Novel HERVs identified in the Kawasaki disease transcriptome database. (**A**) Phylogenetic analysis of the novel HERV-K-2177. The bootstrap values are shown at each branch, with the newly identified viruses highlighted in red font. (**B**) Phylogenetic analysis of the novel HERV-K-3157. The bootstrap values are shown at each branch, with the newly identified viruses highlighted in red font.

To confirm that the newly identified HERV-K-2177 and HERV-K-3157 sequences belong to the HERV family, NJ phylogenetic analyses were conducted on the longer ORFs in which they are located. The analysis included reference sequences from the HERV-K family, assembled from publicly available data from NCBI, as well as major representative ERVs such as *gorilla ERV-K7*, *silver and white gibbon ERV-K7*, and *gopher ERV-K*. Phylogenetic analysis confirmed that the two newly identified genes in the KD transcriptome database (PRJNA271099) are part of the HERV family, clustering with known reference sequences of HERV-K elements with strong bootstrap value support. Both HERV-K-2177 and HERV-K-3157 sequences were distinctly separated from those of other ERVs and clustered in the outermost group of HERV-K family, representing two novel species within the HERV-K family (Fig. 4).

## DISCUSSION

The instability of RNA viruses, along with host diversity and environmental changes, has driven viral mutation and evolution. Retroviruses, which are single-stranded positive-sense RNA viruses infecting vertebrates, integrate into the host genome when infecting germ cells. This integration is inherited vertically from parent to offspring, forming ERVs. Throughout human evolution, exogenous retroviruses have infected human germ cells and integrated into the human genome, giving rise to HERVs, which make up approximately 8% of the human genome. These retroviruses have coexisted with humans for millennia and are closely linked to human development and disease. Although they have only recently been redefined, some HERVs have lost their transcriptional capability due to base insertions or deletions; however, a subset remains regulated by long terminal repeats (LTRs) (33), which can activate a substantial number of transcripts through methylation in immunocompromised or tumorous conditions. These HERVs are implicated in immune disorders, neurological conditions, and neoplasms in humans. Among immune-related diseases, several have been shown to be influenced by HERV transcription, including systemic lupus erythematosus, celiac disease, food allergies, immune thrombocytopenic purpura, herpes simplex, and rheumatoid arthritis (35). HERVs play pivotal roles in cell physiology, immune modulation, and neurodevelopment. For instance, HERV-W encodes the Syncytin-1 protein, which is crucial for placental trophoblast formation and cell fusion (36–38). In hepatocellular carcinoma, Syncytin-1 drives cell cycle progression, migration, and invasion via the MEK/ERK pathway. In leukemia, overexpression of Syncytin-1 inhibits apoptosis and enhances resistance to chemotherapy. HERVs also exhibit dual immunomodulatory functions: activated HERV-K envelope proteins trigger type I interferon responses and the release of pro-inflammatory factors, contributing to the development of autoimmune diseases (39). Conversely, HERV activation in tumors may promote immune escape by inducing antiviral signaling pathways and depleting the IFN pathway (40). Epigenetically regulated HERVs are involved in neuronal RNA transport, and their dysfunction has been associated with neuropsychiatric disorders, such as autism spectrum disorders and schizophrenia, indicating their potential role in disease pathogenesis (41). Over recent decades, advancements in sequencing technology have made it increasingly feasible to generate large-scale genomic data, expanding the understanding of known viruses. However, the number of undiscovered viruses remains far greater than what is currently known. Research on HERVs primarily focuses on known sequences, with limited knowledge about their potential impact on other diseases. Public databases, with sufficient sequencing depth, are now more capable of supporting studies on cells or DNA. The scarcity of research on KD due to limited sample availability has long hindered progress in understanding the connection between viruses and this condition. However, the growing availability of public databases has overcome this challenge, facilitating the identification of novel viruses potentially linked to KD from a broader range of samples.

This study represents the first bioinformatics-based attempt to analyze potential HERV gene transcripts in KD using the public SRA database from NCBI, aiming to identify HERV genes associated with KD onset. A total of 62 pairs of NGS data from children with KD and healthy controls were analyzed, revealing 825 and 5751 differential HERV gene transcripts, with the criteria of |Log_2_FC| > 0.58 and FDR < 0.05 (42). Ferlita et al. identified 130 and 82 dysregulated HERVs in aggressive and inert phenotypes of chronic lymphocytic leukemia (CLL), some of which were specific and validated by RT-PCR. The most upregulated HERV in CLL was highly expressed in samples, and the protein it encodes was positively correlated with Bruton’s tyrosine kinase (BTK) expression in both aggressive and inert CLL phenotypes. Inhibition of HERV expression by treatment with the BTK inhibitor (ibrutinib) led to over 50% survival in the CLL model, suggesting that aberrantly regulated HERVs may be involved in CLL pathogenesis (42). These findings suggest the existence of disease-specific highly expressed HERVs. This preliminary discovery also indicates the abundant diversity of HERVs in the human genome. Additionally, novel HERVs were mined from the KD database by de-joining and splicing the original sequences, extracting retroviruses, annotating, and comparing them. HERV genes encoding amino acid sequences with less than 90% similarity to known sequences were classified as novel. In the PRJNA271099 database, 35 novel HERVs were identified. Two of these, showing upregulated gene expression in the KD group compared to controls, were selected for further investigation. One of these genes, identified as HERV-K18, with an expression level of 4.99, was chosen for the next stage of experimentation.

To validate the expression of HERVs in the peripheral blood of children with KD, blood samples were collected from both patients with KD and healthy controls. Real-time quantitative PCR was employed to assess the differences in the expression of the three HERV genes previously mentioned. The results revealed that HERV-K18 transcript levels were significantly higher in the peripheral blood of children with KD (*P* < 0.05). HERV-K18 encodes a superantigen known to stimulate CD4 +T cells (43), which is associated with the persistence of type 1 diabetes and EBV infection. It is unevenly distributed across different organs, indicating spatio-temporal specificity. *In vitro* experiments have shown that the HERV-K18 superantigen can negatively select human thymocytes, suggesting its role in controlling peripheral tolerance (44). Furthermore, the EBV-encoded latent membrane protein-2A is activated by the immunoreceptor tyrosine-based activation motif (27). Given that EBV infection is commonly associated with the onset of KD in children (45–47), it is essential to explore whether EBV-induced immunosuppression in patients with KD could trigger a substantial activation of HERV-K18 transcriptional superantigen. The potential role of the HERV-K18 superantigen in modulating inflammatory responses and causing peripheral immune tolerance dysregulation warrants further investigation. Additionally, this study found that HERV-K-2177 transcript levels were significantly elevated in the peripheral blood of children with KD compared to healthy controls (*P* < 0.05). In contrast, no significant difference was observed in the transcript levels of HERV-K-3157 (*P* > 0.05). To understand the classification and phylogenetic relationships of novel HERVs, phylogenetic analysis of the two novel HERVs was conducted. The sequences of HERV-K-2177 and HERV-K-3157 were clearly separated from other ERVs, clustering within the HERV-K family and positioned at the outermost group. This indicates that both HERV-K-2177 and HERV-K-3157 represent novel species within the HERV-K family. The HERV-K family has been closely linked to cancer, with its trans-activated transcription of proteins such as env, gag, rec, and NP9 being associated with breast cancer, prostate cancer, and melanoma. These HERV-K proteins contribute to cancer development through mechanisms such as the misregulation of oncogenes, proto-oncogenes, or growth factors by the LTRs of the HERV-K locus (48). The HERV-K family is vast. Accumulating evidence suggests its potential involvement in autoimmune diseases. Oliver Hohn et al. demonstrated that HERV-K gag expression is significantly elevated in the peripheral blood of adult patients with rheumatoid arthritis (RA), contributing to autoimmune responses through molecular mimicry. In adolescents with RA, increased levels of HERV-K superantigens further support a potential link between HERV-K and RA in this population. However, not all members of the HERV-K family are associated with autoimmune diseases; for example, HERV-K113 and HERV-K115 showed no significant increase in the peripheral blood of patients with multiple sclerosis compared to healthy controls (49). In the current study, the expression of HERV-K-2177 was notably higher in the peripheral blood of children with KD, whereas no significant difference was observed for HERV-K-3157, suggesting that different HERV-K members may play distinct roles. Although the precise mechanism behind the elevated expression of HERV-K-2177 in KD pathogenesis was not investigated, studies on other known HERVs have shown that HERV-W can contribute to the development of schizophrenia by inducing cellular pyroptosis, activating innate immune responses, and triggering ferroptosis (50–52). Furthermore, activated HERV-W has been implicated in neurological damage during the pathological progression of multiple sclerosis (53). Additionally, Syncytin-1 plays a significant role in the development of endometrial cancer, leukemia, and other tumors through its involvement in immune evasion mechanisms (22, 37). These findings underscore the pivotal role of HERV-related genes and proteins in the pathogenesis of diseases such as multiple sclerosis, schizophrenia, and various cancers, emphasizing the strong association between HERVs and these conditions. The mechanisms by which HERVs influence KD remain unclear and warrant further investigation.

The present study may further validate the proteins encoded and expressed by the HERV-K family in human coronary artery endothelial cell lines (54), assess cell viability using CCK-8, and measure the expression of KD-associated inflammatory factors (IL-6, IL-8, and NF-κB) via ELISA to explore the relationship between HERVs and the inflammatory response in KD. While protein-level validation was not conducted, the study successfully identified a connection between KD and HERVs.

Nevertheless, several limitations must be acknowledged. The sample size was constrained due to challenges in obtaining rare samples, resulting in the identification of fewer HERVs. Additionally, the study was restricted by time and funding limitations. Despite these constraints, rigorous internal validation methods were employed to ensure the reliability of the data, and the results were consistently replicated across RT-qPCR experiments from different batches, minimizing potential technical artifacts. Although broader generalizations will require larger sample sizes in future studies, this research provides a foundational understanding and sets the stage for subsequent investigations. Future studies will incorporate an increased sample size to validate additional transcriptional features of the HERV gene, thereby further elucidating its role in KD.

## Data Availability

The software codes are publicly available at the following links: https://itol.embl.de/tree/183213198107449561728658121 and https://itol.embl.de/tree/183213198107451441728658129.

## SUPPLEMENTAL MATERIAL

Table S1 spectrum.00189-25-s0001.xlsx Primer sequences after single-peak melting curve screening for qRT-PCR.

Table S2 spectrum.00189-25-s0002.xlsx All protein sequences used for phylogenetic analysis.

ASM does not own the copyrights to Supplemental Material that may be linked to, or accessed through, an article. The authors have granted ASM a non-exclusive, world-wide license to publish the Supplemental Material files. Please contact the corresponding author directly for reuse.
